# Evidence of Weak Habitat Specialisation in Microscopic Animals

**DOI:** 10.1371/journal.pone.0023969

**Published:** 2011-08-24

**Authors:** Diego Fontaneto, Martin Westberg, Joaquín Hortal

**Affiliations:** 1 Silwood Park Campus, Division of Biology, Imperial College London, Ascot Berkshire, United Kingdom; 2 Department of Invertebrate Zoology, Swedish Museum of Natural History, Stockholm, Sweden; 3 Department of Cryptogamic Botany, Swedish Museum of Natural History, Stockholm, Sweden; 4 Departamento de Biodiversidad y Biología Evolutiva, Museo Nacional de Ciencias Naturales, Madrid, Spain; 5 Departamento de Ecologia, Instituto de Ciências Biológicas, Universidade Federal de Goiás, Goiânia, Brazil; Centre National de la Recherche Scientifique, France

## Abstract

Macroecology and biogeography of microscopic organisms (any living organism smaller than 2 mm) are quickly developing into fruitful research areas. Microscopic organisms also offer the potential for testing predictions and models derived from observations on larger organisms due to the feasibility of performing lab and mesocosm experiments. However, more empirical knowledge on the similarities and differences between micro- and macro-organisms is needed to ascertain how much of the results obtained from the former can be generalised to the latter. One potential misconception, based mostly on anedoctal evidence rather than explicit tests, is that microscopic organisms may have wider ecological tolerance and a lower degree of habitat specialisation than large organisms. Here we explicitly test this hypothesis within the framework of metacommunity theory, by studying host specificify in the assemblages of bdelloid rotifers (animals about 350 µm in body length) living in different species of lichens in Sweden. Using several regression-based and ANOVA analyses and controlling for both spatial structure and the kind of substrate the lichen grow over (bark vs rock), we found evidence of significant but weak species-specific associations between bdelloids and lichens, a wide overlap in species composition between lichens, and wide ecological tolerance for most bdelloid species. This confirms that microscopic organisms such as bdelloids have a lower degree of habitat specialisation than larger organisms, although this happens in a complex scenario of ecological processes, where source-sink dynamics and geographic distances seem to have no effect on species composition at the analysed scale.

## Introduction

Although many of the core concepts of ecology have been developed based on experiments and observations of microscopic organisms (e.g. [1], [2]), microbial macroecology and biogeography have been traditionally left out of these advances, failing to provide a consolidated research framework. In fact, the very existence of a biogeography of microscopic organisms is still under debate [3]. Some authors consider that their biogeographical and macroecological patterns are fundamentally different from those of larger organisms; they are small enough to be easily passively dispersed by wind over long distances, they have efficient resting stages allowing them to survive long periods while dormant, and they have asexual and parthenogenetic reproduction which makes it possible for them to rapidly colonise any suitable habitat. This implies that they can be considered mostly cosmopolitan (i.e., the ubiquity hypothesis, that states that for most microbes ‘everything is everywhere, but the environment selects’ [4], [5], [6]). However, many studies on the biogeography and macroecology of microscopic organisms provide evidence of restricted distributions, isolation by distance and geographical gradients, suggesting that many of the processes producing macroecological responses of diversity to area and environmental gradients could be in essence similar in micro- and macroscopic organisms, even if they may differ in scale and magnitude [7], [8], [9]. In other words, there is not enough evidence to support a macroecological and biogeographical dichotomy between micro- and macro-organisms.

Thus, macroecology and biogeography of microscopic organisms may not be different from those of macroscopic ones. Nevertheless, we are far from having a well-supported body of knowledge, and many potential misconceptions are still present in current research on microscopic organisms. The main problem is that the taxonomic diversity in microscopic organisms such as bacteria and protists may be extremely high and difficult to disentangle [10], [11], [12]; thus, masking the spatial patterns and hindering the understanding of the underlying processes. Moreover, the ecological requirements of microbial taxa are difficult to assess and analyse. A good example is given by the presence of thermophilic bacteria in cool temperate soil; are these organisms only waiting in the ‘wrong’ habitat to find a ‘truly suitable’ one, or are their ecological needs so broad as to cover such different habitats [13]?

Microscopic animals can be a more tractable model than bacteria and protists to evaluate the similarities and differences between macro- and micro-organisms. They share size, dispersal abilities, dormancy and asexual reproduction with protists, but have a more approachable diversity. Moreover, they may live in spatially isolated patches, such as lichens, where separate communities of microscopic organisms are present. According to the theoretical framework of metacommunity ecology [14], [15], three alternative predictions can be made for the communities of microscopic animals living in lichen patches: (1) if local species diversity is limited by habitat requirements, then differences in species composition of microscopic organisms should be affected by either the species of lichen, the substrate where lichens grow (due to its physical and chemical effect on the lichen and on the water film around the lichen), or both, but not by geographical distances between samples (i.e., species-sorting metacommunity paradigm); (2) if the species of microorganisms are similar in dispersal and fitness in different habitats, no effect of geographical location or of lichen species or of substrate should be present (i.e., neutral paradigm); and (3) if either source-sink dynamics are important and/or local species diversity is limited by dispersal processes, then differences in species composition should be affected by geographical distances between samples, but not by the species of lichen (i.e., mass-effects or patch dynamics paradigms, respectively).

Here we study the effects of spatial distance and substrate on the occurrence of species and the diversity of communities of bdelloid rotifers living on lichens at a large geographic gradient in Sweden. More specifically, we test the hypothesis that different lichens will show different communities of microscopic organisms, for they produce peculiar sets of chemicals that would influence the water film surrounding them that in turn determines which species are present. This prediction (i.e., that different species of lichen will host different species assemblages) is in fact a strict version of prediction 1. Should we find support for this hypothesis instead of for predictions 2 or 3, our understanding of the ecology of microscopic organisms will be improved by the purging of another misconception, because it would demonstrate that niche partitioning, habitat specialisation and species-sorting processes are acting on micro- as well as on macroscopic organisms [16]. This will support the idea that there is no need for developing independent concepts for microbial macroecology.

**Figure 1:**
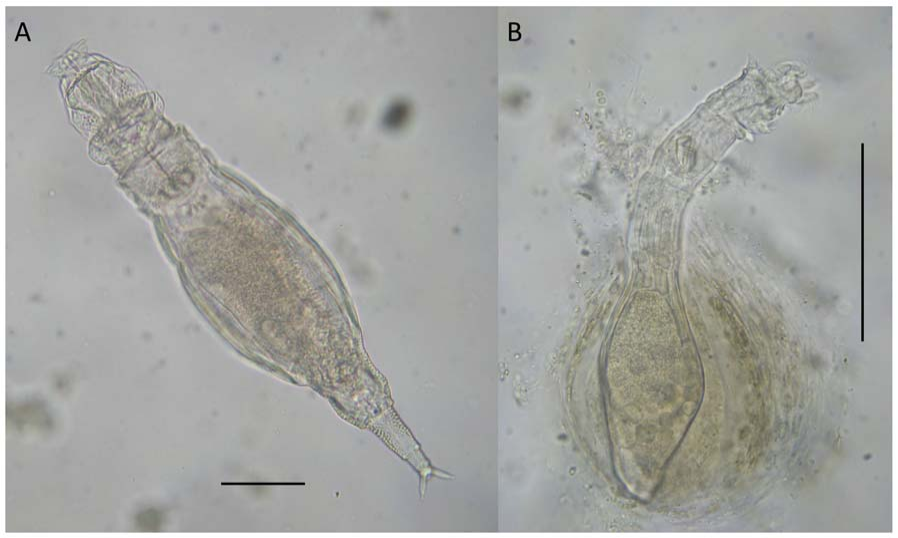
Two of the bdelloid species found in the lichens: A, *Adineta tuberculosa*; B, *Habrotrocha* sp. Scale bar = 0.1 mm. Photo courtesy of Michel Verolet.

Among microscopic organisms living in lichen patches, we focused on bdelloid rotifers (Figure 1), animals with an average body length of 350 µm (range 100–1600 µm), and about 450 species recognised based on morphology only. Even if species complexes are present in bdelloids, each species complex still represents a monophyletic entity, clustering a group of cryptic species with similar ecology [17]; thus, morphological taxonomy may provide reliable estimates of diversity, even if species complexes are present in the system. Bdelloid rotifers are notorious for having a widespread distribution and are considered cosmopolitan, but geographical structure in genetic diversity has been recently described for them [18]. The problem remaining to be solved for bdelloids is determining whether different species have narrow and specific ecological requirements, or whether they can be found in almost any habitat if some minimum requirements are met (e.g., water and food availability). According to the published literature, the latter scenario seems to be the case. Bdelloid rotifers are aquatic and limno-terrestrial animals, and most species have been reported as being able to live in any habitat, from proper water bodies to the water film surrounding soil particles, mosses and lichens [19]. This makes bdelloids a suitable model to test the predictions from the neutral theory [20] among many other theoretical and null models [8], but could as well be a misconception. The environmental conditions that water bodies offer to them cannot be the same found in mosses or lichens; in fact, the species composition of bdelloid assemblages differs significantly between water bodies, mosses and lichens at the local scale [21]. In spite of these differences, all available information suggests that bdelloids do not have any species-specific preference for different moss and/or lichen species [22], [23], [24]. Still, this hypothesis has never been explicitly tested, and all the information is based only on anecdotal reports and indirect evidence.

## Materials and Methods

### Sample collection

**Figure 2:**
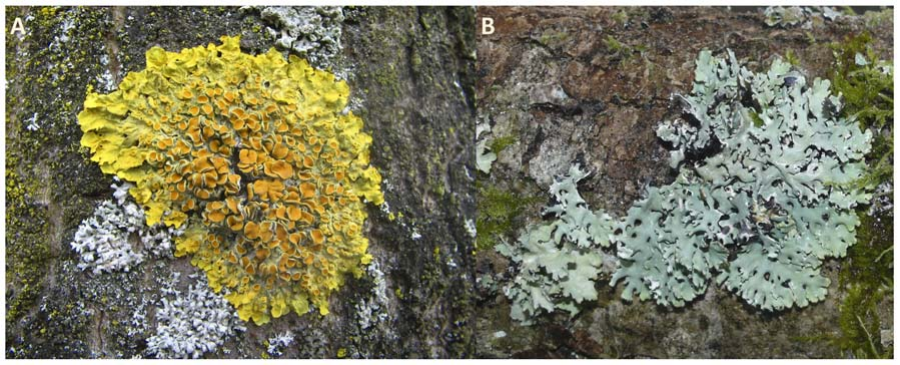
Two of the analysed species of lichen: A, *Xanthoria parietina*; B, *Hypogymnia physodes*. Photos from Wikipedia, freely available under a Creative Commons license (A, http://en.wikipedia.org/wiki/File:Xanthoria_parietina_(06_03_31).jpg; B, http://en.wikipedia.org/wiki/File:Hypogymnia_physodes_010108.jpg).

Ninety-eight lichen samples were collected throughout Sweden, ranging from 55° to 68° North and from 12° to 23° East. No specific permits were required for the described field studies, locations are not privately-owned or protected in any way, and no endangered or protected species were involved. Dry lichen thalli between 5 and 10 cm^2^ were cut from the substrate with a knife, and kept in paper bags. We focused on four foliose lichen species; *Hypogymnia physodes*, *Parmelia saxatilis*, *Parmelia sulcata* and *Xanthoria parietina* (Figure 2). These species offer a rather similar physical habitat for bdelloids, being foliose, similar in size and growing on similar substrates; i.e., all four lichens grow on both siliceous rock substrates and on tree bark. On the other hand, they differ in their ability to retain water due to thallus thickness and the presence of surface structures such as isidia in *P. saxatilis* or soredia in *H. physodes* and *P. sulcata*. The four lichen species also differ in their particular chemistry, both in the cortex and in the medulla. Lichens contain a wide variety of pigments and secondary metabolites serving various functions in the thallus. Cortical substances often function as light-screens, regulating the solar irradiation that reaches the algae symbionts [25]. In this case, *X. parietina* contains anthraquinones (mainly parietin) in the cortex, whereas the other three species have atranorin which belongs to the ß-orcinol *para*-depsides (see [26] and [27] for details on the chemistry of the different lichen species).

For each lichen sample, an area of 2.5 cm^2^ was hydrated with distilled water in a plastic petri dish, whereas the remaining part of the lichen was kept as a voucher in the lichen collection of the Swedish Museum of Natural History. All the active bdelloids that recovered from dormancy in the following four hours after hydration were sorted and identified to species level, following [19]. In order to test for the optimal length of time to look for recovered bdelloids, we performed experiments on additional lichen samples, and we found that bdelloids started recovering between 10 and 40 minutes after hydration of the sample and that no more bdelloids were recovered after four hours. The very few dormant stages still found in the sample that did not recover after that time were always dead. Thus, we are confident that we were able to observe all the species present in the assemblage of each analysed sample.

### Variation in species richness and abundance

We obtained species richness and abundance of individuals for bdelloids living on the lichens and tested whether there were differences in species richness, abundance, composition and preference between lichen species. We also included the substrate of the lichen as a variable (rock vs tree bark) and, when necessary, corrected for the confounding effect of spatial autocorrelation (see below). Each lichen species was sampled as homogeneously as possible throughout the country, in order to minimise spatial autocorrelation, and approximately half of the sample for each lichen species was collected on rocks and half on tree bark (Table S1).

We measured species richness as the observed number of species found in each sample. We cumulated them to obtain the total number of bdelloid species per lichen species. Then, we used linear models (LM) to test whether species richness differed in different lichen species, on different substrate, and the interaction between lichen species and substrate. As species richness data were normally distributed, we used raw data instead of assuming a poisson distribution for count data, which was a worse approximation of the actual distribution. We also tested for the presence of spatial structure, by performing generalized least squares (GLS) models testing the shape of exponential, Gaussian, linear, rational quadratic, and spherical autocorrelation structure [28], using AIC values to select the best model among the non-spatial and spatial ones with all the different correlation structures. All models were performed in R 2.12.0 [29], and GLS with package *nlme* 3.1–97 [30]. Species richness could vary among samples as a mere result of the number of individuals sampled, which could bias in the results. In order to control for such eventual bias, we repeated these analyses after standardising for sample abundance by rarefying all samples to the species richness expected in a subsample of 40 individuals from each species assemblage. Rarefaction calculations were done using the function *rarefy* in vegan, and the seven samples with 40 or less individuals were excluded from these additional analyses.

Abundance of bdelloids was analysed with LM and GLS, as for species richness, but in this case a quasipoisson distribution for count data was used for LM, and a square root transformation was applied for GLS.

### Variation in species composition

For species composition, we excluded the two samples of *X. parietina* without bdelloids; thus, sample size was 96 for this part of the analysis. To analyse the influence of lichen species, substrate, geographical location and their interaction on species composition and partition the variance for each source of variation, we performed a permutational multivariate analysis of variance (*adonis* function in R *vegan* package 1.17–4 [31]) using the matrix of Jaccard distances between species assemblages as response variable. We used raw values of latitude and longitude, but we also tested for non-linear effects of spatial location, by means of the square terms of latitude and longitude. As Jaccard index may be influenced by variation in richness due to variation in abundance of individuals, we repeated the analysis omitting samples with less than 40 and with less than 100 individuals, to check for consistency in the results. If these subsets with only samples with a high number of individuals provide qualitatively similar results, we can be confident about the strength and generality of the hypothesis; alternatively, if results are not consistent, drivers influencing abundance indirectly influence richness and we would need to disentangle them.

As a further test on the effect of geographic location of samples on community composition, we tested for the presence of significant distance-decay scenarios, using Mantel tests between geographic distances and distances in community composition (Jaccard index) on all samples and on each lichen species separately.

We assessed the effects of both lichen species and substrate on the abundance of single species of bdelloids using both Multiple Factorial ANOVA and Partial Least Squares regression analyses (PLS). Given the lack of spatial structure in the data (see results), the geographical location of the samples was not taken into account in these analyses, thus simplifying the design of hypotheses. Also, all species found only once were discarded. We ran Factorial ANOVAs using the abundance of each species as dependent variable and lichen species and substrate as factors, assessing their significance in a multivariate test by means of Wilks' *Lambda*. PLS are particularly well suited to analyzing a large array of dependent and independent variables with a limited sample size, avoiding also problems of multicolinearity [32]. Briefly, PLS is an extension of multiple regression analysis where both predictors and dependent variables (when there is more than one) are first transformed into latent factors, that is, a lower number of orthogonal factors that are extracted as linear combinations of these variables. These latent factors are used to establish associations between the sets of dependent variables (set Y) and predictors (set X), providing measures of the amount of variability of each one of these sets that is explained by the overall structure of the data [32], [33], [34].

## Results

A total of 62 bdelloid species (Figure 1) was found in the 98 lichen samples (Table S2). Model fit through AIC for all models of species richness and abundance indicated that spatial models were not significantly better than the non-spatial ones, neither for species richness nor for abundance. Visual inspection of the shape of the variograms also confirmed that spatial autocorrelation was not present. The results of the GLS models always matched qualitatively and quantitatively the results from the linear models. Thus, we show LM results, which have no spatial structure and are simpler in their interpretation. Species richness was significantly lower in *X. parietina* (median 4, range 0–8) than in the other lichen species (medians 5–6, ranges 3–11) (Linear Model: t = −2.21, p = 0.028), whereas no effect of substrate was detected. The results based on rarefied richness scores were qualitatively similar, with no effect of substrate on species richness of bdelloids and significantly less species in *X. parietina* (Linear Model: t = −2.607, p = 0.011), an effect that may be even stronger if we take into account that five out of the seven samples that were omitted for this analysis because they had less than 40 individuals pertained to this lichen species.

Abundance of bdelloids was not different between lichen species, whereas lichens on rocks hosted significantly more specimens of bdelloids (median 152, range 0–347) than those on tree barks (median 85, range 0–180) (Linear Model: t = 2.43, p = 0.017).

Species composition of bdelloids was significantly influenced by the species of lichen, which explained 11% of the variance. The kind of substrate, the geographic location and their interactions also explained additional significant portions of the variability in species composition, although as much as 77% of the variance was not explained by the analysed variables (Table 1). The results were qualitatively similar when controlling for differences in abundance by including only species assemblages with at least 40 or at least 100 individuals (Table S3). We did not find any relationship between geographic distance of samples and differences in community composition, neither for the whole dataset, nor for each of the four lichen species separately (Mantel test: r values between 0.01 and 0.05; all p values≫0.05).

**Table 1:** Results of the permutational multivariate analysis of variance on Jaccard distances between species compositions on each lichen sample, retaining only the significant terms and interactions.

Variable	Df	R^2^	p
lichen	3	0.1179	0.001
substrate	1	0.0185	0.005
lichen∶substrate	3	0.0415	0.006
latitude	1	0.0169	0.009
latitude^2^	1	0.0164	0.017
longitude	1	0.0141	0.041
residuals	85	0.7746	

Overall, bdelloid species showed habitat selection according to the Multiple Factorial ANOVA analyses; both substrate and lichen species, as well as their interaction, had a significant effect on the abundance of bdelloid species (Table 2). However, only nineteen out of the fifty species analyzed showed significant habitat selection according to the single-species ANOVA analyses (Table S4). This was evidenced by the relatively low variability in the dependent variables (i.e., species abundances) explained by the PLS analysis, where the seven latent factors extracted were able to account for only 15% of the variation in species abundances (Table 3). Nevertheless, for more than half of the species one or several latent factors were able to explain more than 10% of the variability of abundances each (Table S4), evidencing either positive or negative effects of the lichen species and/or the substrate, and thus significant habitat selection. Among these bdelloid species, *Adineta tuberculosa*, *Adineta vaga*, *Ceratotrocha cornigera*, *Didymodactylos carnosus*, *Habrotrocha spicula*, *Habrotrocha pulchra*, *Habrotrocha* sp. 3, *Mniobia incrassata*, *Mniobia scarlatina* and *Philodina proterva* showed the highest levels of habitat selection, which in general were related to more than one of the latent factors identified by the PLS analysis (Table S4). Interestingly, all these species but *H. pulchra* showed negative relationships with the lichen species *Hypogymnia physodes*. Similarly, only *P. proterva* was not negatively related with tree bark (Table S1).

**Table 2:** Overall results of the Multiple Factorial ANOVA analyzing the individual responses of each bdelloid species to lichen species, substrate, and their interaction.

	Wilk's Lambda	df (effect,error)	F
intercept	0.0232	50,39	32.85
lichen	0.0095	150,117.83	2.93
substrate	0.2112	50,39	2.91
lichen∶substrate	0.0238	150,117.83	1.95

The Wilk's Lambda statistic measures the multivariate association between these factors throughout all bdelloid species, and its significance is assessed by means of the F statistic; all effects were significant to p<0.001.

**Table 3:** Results of the Partial Least Squares Analysis.

	Explained variability		Predictor weights						
	Avg.R^2^ of Y	Avg.R^2^ of X	Hphy	Psax	Psul	bark	Hphy: bark	Psax: bark	Psul: bark
LF1	0.052	0.264	0.412	0.709	0.505	−0.166	−0.081	−0.160	−0.118
LF2	0.077	0.520	−0.463	0.297	−0.121	−0.578	−0.424	−0.291	−0.291
LF3	0.092	0.713	−0.352	0.350	−0.157	−0.622	0.270	0.297	0.426
LF4	0.111	0.799	−0.123	−0.305	0.548	−0.007	−0.581	0.501	0.051
LF5	0.130	0.868	−0.409	0.547	−0.112	0.289	0.059	0.482	−0.448
LF6	0.144	0.939	0.158	−0.350	0.219	−0.394	0.346	0.297	−0.664
LF7	0.153	1.000	−0.565	−0.102	0.574	0.038	0.478	−0.315	−0.109

Avg.R^2^ is the average amount of variability explained by the combinations of one to seven latent factors (LF1-7), Latent factors are formed by linear combinations of all dependent variables (i.e., species) (Y) and predictors (X); the weights of the latter on each of these factors are also quoted. Predictor codes are for three species of lichen (Hphy – *Hypogymnia physodes*, Psax – *Parmelia saxatilis*, Psul – *P. sulcata*) and one substrate category (bark – tree bark), that are the qualitative states of these two ordinary variables; note that *Xanthoria parietina* and rock are redundant in the codification and are thus not included in the analyses.

## Discussion

The first interpretation of our results is straightforward: notwithstanding wide ecological tolerance with most bdelloid species present in different lichens, several species of bdelloids have significantly different preferences for lichen species and for the substrate. This means that species sorting processes have a significant effect on the composition of bdelloid assemblages living on lichens, providing support for our general prediction, which corresponds to our prediction 1, that is, the existence of species-sorting processes, according to [14]. At the same time, a large overlap in species composition between lichens and types of substrate is present, suggesting that such preferences are not strict. However, these compositional similarities are not the product of source-sink dynamics, because the geographical proximity between samples does not have any impact on the composition of their bdelloid assemblages; in fact, at the scale of the present study no distance-decay in species composition is present, which allows us to reject prediction 3 on mass effects and/or patch dynamics (see also [35]).

The interpretation of our results is, however, not so simple in what refers to prediction 2 (i.e., neutral metacommunity processes). Neutral dynamics are often associated to geographically-structured similarities among assemblages due to their reliance on dispersal processes [15], which are often distance-limited for macrobes. However, bdelloids are known to have much wider geographical ranges than macroscopic organisms and to have a high ratio of long-distance processes [18], [36]. If we take this key characteristic into account (see discussion in [8]), the combination of limited habitat specialisation and lack of geographical structure could be interpreted as being the effect of a balance between species-sorting and neutral processes (i.e., both predictions 1 and 2 are partially met). In other words, the composition of bdelloid assemblages would be driven by a mixture of neutral dynamics with no apparent dispersal limitation at the scale considered and a limited but significant specialisation of many species adapted to dwell over certain lichen species and/or substrate. Therefore, we argue that the differences in the assemblages of bdelloid rotifers living on Swedish lichens are the result of stochastic environmental variations, large dispersal ability and wide ecological tolerances.

An important aspect of our results is that both the old empirical suggestion that microscopic organisms such as bdelloids have wider ecological tolerance than macroscopic ones and our hypothesis that also microscopic organisms have habitat specialisation are partially met. Critically, we find evidence that the degree of habitat specialisation in a large number of bdelloid species is low, perhaps lower than in many macroscopic organisms. For example, phytophagous insects (e.g., butterflies, fig wasps and fruitflies) are all strictly linked to the habitat where their host plants are present, and most of them can feed only on a very narrow range of plants [37], [38], [39]. Bdelloids do not show such strong specialisation, but the only two samples of lichens without bdelloids were collected on rocks close to the sea, and it is known that very few bdelloids can cope with saltwater [40]. However, it is important to note that high habitat specificity (i.e., narrow niche width) is also not general for many macroscopic organisms (see discussion in [41]). Thus, although the degree of habitat specialisation seems apparently lower in microorganisms, further studies are necessary to determine if these differences are significant when a more comprehensive set of macrobes and microbes are taken into account.

A similar situation of low degree of habitat specialisation is present in most microscopic animals, for example, in gastrotrichs and in tardigrades [42], [43]. Unfortunately, whether this potentially wide ecological tolerance is the actual scenario or an artifact of our inability to describe their ecological requirements cannot be decided with our results. No previous explicit study has yet been performed, and our analysis of habitat specialisation of bdelloids in lichens has mixed results. Still, at the very local scale, it is known that epibiont rotifers living on a freshwater crustacean show strong preferences for their spatial localisation on the host and that different species compete and interact for space [16]. At the landscape scale, bdelloids assemblages in lichens, mosses and water bodies are significanlty different in their species composition [21], and species richness is affected by altitude in planktonic rotifers [44]. The apparent absence of a strong separation in the habitat requirements of the analysed lichen-dwelling bdelloids may thus be an artifact of our definition of habitat specificity. Still, although we did not measure directly any ecological variable, we used different species of lichens which differ in most of the ecological aspects relevant to bdelloids, such as rate of evapotraspiration, chemical composition or physical structure. In spite of these arguably important differences, in our study bdelloid species show only weak preferences for different lichen species.

It could be argued that being strictly parthenogenetic (and thus asexual) organisms, bdelloids could be expected to have wide ecological tolerances linked to a so-called ‘general purpose genotype’ that may allow them to survive notwithstanding the evolutionary disadvantages of lacking sexual recombination [45]. However, parthenogenetic organisms may show also ‘frozen niche’ [46], [47], and the origin of their wide tolerances may be more complex. In fact, most microscopic animals such as bdelloid rotifers, gastrotrichs and tardigrades show a similar degree of ecological tolerance and wide geographical distribution in spite of their different reproductive modes, from obligate to facultative parthenogenesis and only sexual reproduction [3]. Thus, we may confidently reject the hypothesis that wide ecological tolerance is linked to bdelloid asexuality. Yet, as bdelloids are completely asexual, the driving force promoting speciation in this group can only be ecological specialisation, as reproductive isolation does not exert any significant impact on their evolution [48]. This leads to the paradox that the origin and presence of different bdelloid species is not accompanied by clear differences in habitat preferences, but rather by an apparently wide ecological overlap between lichen-dwelling species. Nevertheless, almost nothing is known on the mechanisms promoting speciation in bdelloids, and this phenomenon may as well happen as a result of the co-occurrence of general purpose genopyes.

Our main conclusion is that some microscopic organisms such as bdelloids share relatively similar ecological patterns and processes with macroscopic organisms, in spite of some important differences in, e.g., their dispersal ability. Thus, we support the idea that there is no need for developing independent concepts for microbial macroecology. On the contrary, the existing similarities may make microscopic animals useful study subjects in the developing framework of experimental biogeography (see [8], [9]). Still, more empirical field studies and experiments (e.g., [35], [49]) need to be performed to be able to provide suitable comparison between micro- and macro-organisms and to be able to obtain useful generalisations from these unconspicuous, little known organisms.

## Supporting Information

Table S1 **Lichen samples used in the analysis.**
(DOCX)

Table S2 **Bdelloid species found in the four lichen species.** Species named sp. followed by a number refer to unknown morphotypes, potentially new, still undescribed species.
(DOCX)

Table S3 **Results of the permutational multivariate analysis of variance on Jaccard distances between species compositions on each lichen sample, retaining the same significant terms and interactions of** **Table 1** **, performed on two reduced datasets, including only samples with at least 40 individuals (N = 91), and at least 100 individuals (N = 50).**
(DOCX)

Table S4 **Results of the Factorial ANOVA and PLS analyses relating the abundance of single species of bdelloids with lichen species and substrate.** Species with significant ANOVA relationships are highlighted in bold. Note that 13 species that had only one occurrence have been excluded from these analyses.
(DOCX)
