# Supplementary material for: Evidence of Weak Habitat Specialisation in Microscopic Animals
Source: PLoS One. 2011 Aug 24;6(8):e23969. doi: 10.1371/journal.pone.0023969 (PMC3161089; doi:10.1371/journal.pone.0023969)
Supplement: Table S1 — Lichen samples used in the analysis. (DOCX) [file pone.0023969.s001.docx]

**Table S1.** Lichen samples used in the analysis.

| ID | Lichen species | substrate | North | East |
| --- | --- | --- | --- | --- |
| A627 | *Hypogymnia physodes* | bark | 65.98333 | 19.34534 |
| A625 | *Hypogymnia physodes* | bark | 65.95555 | 19.51461 |
| A632 | *Hypogymnia physodes* | bark | 65.95268 | 19.82504 |
| A623 | *Hypogymnia physodes* | bark | 65.71989 | 23.07758 |
| A636 | *Hypogymnia physodes* | bark | 65.61603 | 21.41402 |
| A639 | *Hypogymnia physodes* | bark | 65.56588 | 21.46791 |
| A634 | *Hypogymnia physodes* | bark | 65.5341 | 18.98805 |
| A549 | *Hypogymnia physodes* | bark | 59.2779 | 18.7985 |
| A546 | *Hypogymnia physodes* | bark | 59.27779 | 18.79448 |
| A529 | *Hypogymnia physodes* | bark | 59.26445 | 18.7665 |
| A530 | *Hypogymnia physodes* | bark | 59.26217 | 18.75625 |
| A655 | *Hypogymnia physodes* | bark | 58.82141 | 12.48092 |
| A652 | *Hypogymnia physodes* | bark | 58.80655 | 12.42277 |
| A650 | *Hypogymnia physodes* | bark | 58.8061 | 12.43664 |
| A659 | *Hypogymnia physodes* | bark | 58.78025 | 12.42672 |
| A611 | *Hypogymnia physodes* | bark | 57.72264 | 16.41883 |
| A613 | *Hypogymnia physodes* | bark | 57.71313 | 16.58113 |
| A614 | *Hypogymnia physodes* | bark | 57.71313 | 16.58112 |
| A610 | *Hypogymnia physodes* | bark | 57.67748 | 16.42909 |
| A644 | *Hypogymnia physodes* | bark | 55.70452 | 13.20319 |
| A520 | *Hypogymnia physodes* | bark | 55.67708 | 13.4052 |
| A510 | *Hypogymnia physodes* | bark | 55.56694 | 13.21195 |
| A678 | *Hypogymnia physodes* | rock | 60.21985 | 18.727 |
| A773 | *Hypogymnia physodes* | rock | 60.15564 | 14.96842 |
| A679 | *Hypogymnia physodes* | rock | 60.02793 | 18.85041 |
| A680 | *Hypogymnia physodes* | rock | 59.88955 | 17.7513 |
| A681 | *Hypogymnia physodes* | rock | 59.88287 | 17.78627 |
| A677 | *Hypogymnia physodes* | rock | 59.27913 | 18.77155 |
| A658 | *Hypogymnia physodes* | rock | 58.8214 | 12.48091 |
| A612 | *Hypogymnia physodes* | rock | 57.76237 | 16.68762 |
| A700 | *Hypogymnia physodes* | rock | 56.1151 | 14.68155 |
| A701 | *Hypogymnia physodes* | rock | 56.11464 | 14.68025 |
| A760 | *Parmelia saxatilis* | bark | 63.80259 | 12.83118 |
| A764 | *Parmelia saxatilis* | bark | 63.80237 | 12.82401 |
| A770 | *Parmelia saxatilis* | bark | 61.37766 | 14.82399 |
| A771 | *Parmelia saxatilis* | bark | 61.37619 | 14.82348 |
| A686 | *Parmelia saxatilis* | bark | 59.96442 | 17.3169 |
| A762 | *Parmelia saxatilis* | bark | 58.89227 | 12.3794 |
| A646 | *Parmelia saxatilis* | bark | 58.8062 | 12.43664 |
| A668 | *Parmelia saxatilis* | bark | 58.35421 | 11.93585 |
| A685 | *Parmelia saxatilis* | bark | 58.33325 | 11.92444 |
| A628 | *Parmelia saxatilis* | rock | 65.98334 | 19.34533 |
| A631 | *Parmelia saxatilis* | rock | 65.95269 | 19.82504 |
| A621 | *Parmelia saxatilis* | rock | 65.71989 | 23.07759 |
| A640 | *Parmelia saxatilis* | rock | 65.58528 | 21.41383 |
| A671 | *Parmelia saxatilis* | rock | 64.84673 | 23.48044 |
| A550 | *Parmelia saxatilis* | rock | 59.2778 | 18.7985 |
| A545 | *Parmelia saxatilis* | rock | 59.27779 | 18.79449 |
| A512 | *Parmelia saxatilis* | rock | 59.264867 | 18.770367 |
| A528 | *Parmelia saxatilis* | rock | 59.25342 | 18.76347 |
| A654 | *Parmelia saxatilis* | rock | 58.8214 | 12.48092 |
| A656 | *Parmelia saxatilis* | rock | 58.818 | 12.48092 |
| A645 | *Parmelia saxatilis* | rock | 58.81726 | 12.43979 |
| A618 | *Parmelia saxatilis* | rock | 57.76238 | 16.68763 |
| A617 | *Parmelia saxatilis* | rock | 57.72263 | 16.41883 |
| A619 | *Parmelia saxatilis* | rock | 57.67748 | 16.42908 |
| A633 | *Parmelia sulcata* | bark | 65.96302 | 19.81367 |
| A624 | *Parmelia sulcata* | bark | 65.95555 | 19.51462 |
| A622 | *Parmelia sulcata* | bark | 65.71988 | 23.07759 |
| A638 | *Parmelia sulcata* | bark | 65.61602 | 21.41401 |
| A525 | *Parmelia sulcata* | bark | 59.37071 | 18.05274 |
| A648 | *Parmelia sulcata* | bark | 58.80655 | 12.42278 |
| A653 | *Parmelia sulcata* | bark | 58.78249 | 12.42678 |
| A657 | *Parmelia sulcata* | bark | 58.78026 | 12.42671 |
| A669 | *Parmelia sulcata* | bark | 58.3542 | 11.93585 |
| A616 | *Parmelia sulcata* | bark | 57.67749 | 16.42909 |
| A641 | *Parmelia sulcata* | bark | 55.70452 | 13.20318 |
| A511 | *Parmelia sulcata* | bark | 55.56694 | 13.21196 |
| A761 | *Parmelia sulcata* | rock | 63.80393 | 12.7883 |
| A772 | *Parmelia sulcata* | rock | 60.15565 | 14.96842 |
| A688 | *Parmelia sulcata* | rock | 60.02792 | 18.85041 |
| A687 | *Parmelia sulcata* | rock | 59.88286 | 17.78627 |
| A676 | *Parmelia sulcata* | rock | 59.27913 | 18.77156 |
| A763 | *Parmelia sulcata* | rock | 58.89228 | 12.3794 |
| A615 | *Parmelia sulcata* | rock | 57.76238 | 16.68762 |
| A620 | *Xanthoria parietina* | bark | 65.72483 | 23.07915 |
| A637 | *Xanthoria parietina* | bark | 65.61602 | 21.41402 |
| A524 | *Xanthoria parietina* | bark | 59.37555 | 18.04705 |
| A526 | *Xanthoria parietina* | bark | 59.37071 | 18.05273 |
| A527 | *Xanthoria parietina* | bark | 59.36823 | 18.05484 |
| A649 | *Xanthoria parietina* | bark | 58.80656 | 12.42278 |
| A604 | *Xanthoria parietina* | bark | 57.73761 | 16.42045 |
| A606 | *Xanthoria parietina* | bark | 57.72442 | 16.42909 |
| A605 | *Xanthoria parietina* | bark | 57.71616 | 16.614 |
| A507 | *Xanthoria parietina* | bark | 55.80085 | 13.37015 |
| A642 | *Xanthoria parietina* | bark | 55.70453 | 13.20318 |
| A643 | *Xanthoria parietina* | bark | 55.70453 | 13.20319 |
| A506 | *Xanthoria parietina* | bark | 55.56694 | 13.21194 |
| A769 | *Xanthoria parietina* | rock | 68.35205 | 18.81674 |
| A698 | *Xanthoria parietina* | rock | 59.89805 | 17.63357 |
| A699 | *Xanthoria parietina* | rock | 59.36786 | 18.05541 |
| A551 | *Xanthoria parietina* | rock | 59.2779 | 18.7986 |
| A683 | *Xanthoria parietina* | rock | 58.71997 | 12.47171 |
| A768 | *Xanthoria parietina* | rock | 57.3618 | 17.1171 |
| A766 | *Xanthoria parietina* | rock | 57.36107 | 17.11824 |
| A767 | *Xanthoria parietina* | rock | 57.36079 | 17.11618 |
| A505 | *Xanthoria parietina* | rock | 55.66393 | 13.34576 |
