# Supplementary material for: Evidence of Weak Habitat Specialisation in Microscopic Animals
Source: PLoS One. 2011 Aug 24;6(8):e23969. doi: 10.1371/journal.pone.0023969 (PMC3161089; doi:10.1371/journal.pone.0023969)
Supplement: Table S2 — Bdelloid species found in the four lichen species. Species named sp. followed by a number refer to unknown morphotypes, potentially new, still undescribed species. (DOCX) [file pone.0023969.s002.docx]

**Table S2.** Bdelloid species found in the four lichen species. Species named sp. followed by a number refer to unknown morphotypes, potentially new, still undescribed species.

|  | *Hypogymnia physodes* | *Parmelia saxatilis* | *Parmelia sulcata* | *Xanthoria parietina* |
| --- | --- | --- | --- | --- |
| *Adineta barbata* Janson, 1893 | X |  | X |  |
| *Adineta cuneata* Milne, 1916 | X | X | X |  |
| *Adineta elongata* Rodewald, 1935 |  |  | X |  |
| *Adineta gracilis* Janson, 1893 | X | X | X |  |
| *Adineta steineri* Bartos, 1951 | X |  | X |  |
| *Adineta tuberculosa* Janson, 1893 | X | X | X |  |
| *Adineta vaga* (Davis, 1873) | X |  | X | X |
| *Ceratotrocha cornigera* (Bryce, 1893) |  | X | X |  |
| *Ceratotrocha franzi* Donner, 1949 | X |  |  |  |
| *Didymodactyos carnosus* Milne, 1916 | X | X | X |  |
| *Habrtrocha bidens* (Gosse, 1851) | X | X | X | X |
| *Habrotrocha constricta* (Dujardin, 1841) | X | X | X | X |
| *Habrotrocha crenata* (Murray, 1905) |  | X |  |  |
| *Habrotrocha elusa* Milne, 1916 |  |  | X | X |
| *Habrotrocha ligula* Bryce, 1913 | X | X | X | X |
| *Habrotrocha pulchra* (Murray, 1905) | X | X | X | X |
| *Habrotrocha pusilla nuda* Donner, 1950 | X |  | X | X |
| *Habrotrocha quinquedens* de Koning, 1947 | X |  |  |  |
| *Habrotrocha rosa* Donner, 1949 | X |  |  |  |
| *Habrotrocha spicula* Bryce, 1913 | X | X | X |  |
| *Habrotrocha sylvestris* Bryce, 1915 | X |  | X |  |
| *Habrotrocha* sp. 1 | X | X | X |  |
| *Habrotrocha* sp. 2 |  | X | X |  |
| *Habrotrocha* sp. 3 |  |  |  | X |
| *Habrotrocha tridens* (Milne, 1886) |  | X |  | X |
| *Macrotrachela decora* (Bryce, 1912) | X |  |  |  |
| *Macrotrachela ehrenbergi* (Janson, 1893) | X | X | X |  |
| *Macrotrachela habita* (Bryce, 1894) | X | X | X | X |
| *Macrotrachela insolita* de Koning, 1947 | X |  | X | X |
| *Macrotrachela musculosa* (Milne,, 1886) | X | X | X | X |
| *Macrotrachela papillosa* (Thompson, 1892) | X | X | X |  |
| *Macrotrachela plicata plicata* (Bryce, 1892) | X | X | X | X |
| *Macrotrachela plicata hirundinella* (Murray, 1905) | X | X | X |  |
| *Macrotrachela punctata* (Murray, 1911) | X | X | X |  |
| *Macrotrachela quadricornifera* Milne, 1886 |  | X | X | X |
| *Mniobia bredensis* de Koning, 1947 | X | X |  | X |
| *Mniobia incrassata* (Murray, 1905) | X | X |  |  |
| *Mniobia magna* (Plate, 1889) | X | X | X | X |
| *Mniobia montium* Murray, 1911 | X | X |  | X |
| *Mniobia obtusicalcar* de Koning, 1947 | X |  |  | X |
| *Mniobia obtusicornis* Murray, 1911 | X |  | X |  |
| *Mniobia recurvicornis* Bartos, 1950 |  |  | X | X |
| *Mniobia russeola* (Zelinka, 1891) | X | X | X | X |
| *Mniobia scabrosa* Murray, 1911 | X | X | X |  |
| *Mniobia scarlatina* (Ehrenberg, 1853) | X | X | X | X |
| *Mniobia tentans* Donner, 1949 | X | X | X | X |
| *Mniobia* sp. 1 | X |  | X |  |
| *Mniobia* sp. 2 | X |  |  | X |
| *Otostephanos torquatus* (Bryce, 1913) | X |  | X |  |
| *Otostephanos* sp. 1 |  | X |  |  |
| *Pleuretra humerosa* (Murray, 1905) |  | X |  |  |
| *Pleuretra lineata* Donner, 1962 |  | X |  |  |
| *Pleuretra* sp. 1 | X | X | X |  |
| *Philodina morigera* Donner, 1949 | X |  |  |  |
| *Philodina plena* (Bryce, 1894) | X |  |  | X |
| *Philodina proterva* Milne, 1916 | X |  | X | X |
| *Philodina rugosa* Bryce, 1903 | X | X | X |  |
| *Philodina* sp. 1 | X |  |  |  |
| *Philodina* sp. 2 |  |  |  | X |
| *Philodina vorax* (Janson, 1893) |  |  | X | X |
| *Scepanotrocha corniculata* (Bryce, 1910) |  | X |  |  |
| *Scepanotrocha* sp. 1 | X |  | X |  |
