# Supplementary material for: Evidence of Weak Habitat Specialisation in Microscopic Animals
Source: PLoS One. 2011 Aug 24;6(8):e23969. doi: 10.1371/journal.pone.0023969 (PMC3161089; doi:10.1371/journal.pone.0023969)
Supplement: Table S3 — Results of the permutational multivariate analysis of variance on Jaccard distances between species compositions on each lichen sample, retaining the same significant terms and interactions of Table 1 , performed on two reduced datasets, including only samples with at least 40 individuals (N = 91), and at least 100 individuals (N = 50). (DOCX) [file pone.0023969.s003.docx]

**Table S3.** Results of the permutational multivariate analysis of variance on Jaccard distances between species compositions on each lichen sample, retaining the same significant terms and interactions of Table 1, performed on two reduced datasets, including only samples with at least 40 individuals (N = 91), and at least 100 individuals (N = 50).

| Variable | df_(40)_ | R^2^_(40)_ | P_(40)_ | df_(100)_ | R^2^_(100)_ | P_(100)_ |
| --- | --- | --- | --- | --- | --- | --- |
| lichen | 3 | 0.1275 | 0.001 | 3 | 0.1395 | 0.001 |
| substrate | 1 | 0.0182 | 0.009 | 1 | 0.0374 | 0.006 |
| lichen:substrate | 3 | 0.0410 | 0.010 | 3 | 0.0636 | 0.101 |
| latitude | 1 | 0.0163 | 0.025 | 1 | 0.0213 | 0.213 |
| latitude^2^ | 1 | 0.0140 | 0.085 | 1 | 0.0259 | 0.065 |
| longitude | 1 | 0.0139 | 0.067 | 1 | 0.0236 | 0.118 |
| residuals | 80 | 0.7690 |  | 39 | 0.6885 |  |
