# Supplementary material for: Evidence of Weak Habitat Specialisation in Microscopic Animals
Source: PLoS One. 2011 Aug 24;6(8):e23969. doi: 10.1371/journal.pone.0023969 (PMC3161089; doi:10.1371/journal.pone.0023969)
Supplement: Table S4 — Results of the Factorial ANOVA and PLS analyses relating the abundance of single species of bdelloids with lichen species and substrate. Species with significant ANOVA relationships are highlighted in bold. Note that 13 species that had only one occurrence have been excluded from these analyses. (DOCX) [file pone.0023969.s004.docx]

**Table S4.** Results of the Factorial ANOVA and PLS analyses relating the abundance of single species of bdelloids with lichen species and substrate. Species with significant ANOVA relationships are highlighted in bold. Note that 13 species that had only one occurrence have been excluded from these analyses.

|  | ANOVA |  |  |  | PLS R^2^ per latent factor | | | | | | |  | PLS scaled regression coefficients | | | | | | |
| --- | --- | --- | --- | --- | --- | --- | --- | --- | --- | --- | --- | --- | --- | --- | --- | --- | --- | --- | --- |
| Species | Adj.R^2^ | F | p |  | LF1 | LF2 | LF3 | LF4 | LF5 | LF6 | LF7 |  | Hphy | Psax | Psul | bark | Hphy:bark | Psax:bark | Psul:bark |
| *Adineta barbata* | 0.006 | 1.09 | 0.380 |  | 0.005 | 0.017 | 0.022 | 0.024 | 0.024 | 0.073 | 0.079 |  | 0.069 | -0.115 | 0.158 | -0.037 | 0.223 | 0.025 | -0.244 |
| *Adineta cuneata* | 0.006 | 1.08 | 0.381 |  | 0.014 | 0.039 | 0.043 | 0.054 | 0.057 | 0.067 | 0.079 |  | 0.088 | -0.064 | 0.133 | 0.024 | 0.242 | -0.110 | -0.091 |
| ***Adineta gracilis*** | **0.099** | **2.49** | **0.022** |  | **0.033** | **0.070** | **0.070** | **0.094** | **0.102** | **0.163** | **0.165** |  | **0.101** | **-0.105** | **0.215** | **-0.309** | **-0.102** | **0.104** | **-0.215** |
| ***Adineta steineri*** | **0.115** | **2.76** | **0.012** |  | **0.012** | **0.012** | **0.014** | **0.022** | **0.024** | **0.146** | **0.180** |  | **-0.080** | **-0.163** | **0.381** | **-0.170** | **0.226** | **0.117** | **-0.424** |
| ***Adineta tuberculosa*** | **0.347** | **8.22** | **<0.001** |  | **0.155** | **0.325** | **0.348** | **0.348** | **0.374** | **0.392** | **0.395** |  | **-0.006** | **0.148** | **0.212** | **-0.471** | **-0.034** | **-0.183** | **-0.105** |
| ***Adineta vaga*** | **0.422** | **10.92** | **<0.001** |  | **0.384** | **0.385** | **0.448** | **0.452** | **0.464** | **0.465** | **0.465** |  | **-0.244** | **-0.355** | **-0.196** | **-0.127** | **0.042** | **0.087** | **0.227** |
| ***Ceratotrocha cornigera*** | **0.219** | **4.80** | **<0.001** |  | **0.069** | **0.169** | **0.196** | **0.247** | **0.248** | **0.249** | **0.276** |  | **-0.192** | **0.328** | **0.024** | **-0.255** | **0.192** | **-0.325** | **-0.024** |
| ***Didymodactyos carnosus*** | **0.205** | **4.50** | **<0.001** |  | **0.114** | **0.155** | **0.188** | **0.189** | **0.225** | **0.251** | **0.264** |  | **-0.287** | **0.493** | **0.120** | **-0.062** | **-0.051** | **-0.007** | **0.010** |
| *Habrtrocha bidens* | 0.011 | 1.15 | 0.340 |  | 0.000 | 0.001 | 0.014 | 0.032 | 0.041 | 0.048 | 0.084 |  | 0.093 | 0.057 | -0.110 | 0.136 | -0.311 | 0.179 | 0.008 |
| *Habrotrocha constricta elusa* | 0.005 | 1.07 | 0.389 |  | 0.042 | 0.050 | 0.055 | 0.057 | 0.073 | 0.074 | 0.079 |  | 0.022 | -0.127 | -0.178 | -0.098 | -0.045 | -0.074 | 0.144 |
| *Habrotrocha elusa* | 0.053 | 1.76 | 0.105 |  | 0.006 | 0.012 | 0.012 | 0.044 | 0.044 | 0.079 | 0.123 |  | -0.233 | -0.215 | 0.338 | -0.131 | 0.099 | 0.090 | -0.211 |
| *Habrotrocha ligula* | 0.062 | 1.89 | 0.080 |  | 0.003 | 0.073 | 0.080 | 0.087 | 0.087 | 0.088 | 0.131 |  | -0.218 | 0.057 | 0.140 | -0.082 | 0.115 | -0.248 | -0.153 |
| ***Habrotrocha pulchra*** | **0.161** | **3.60** | **0.002** |  | **0.164** | **0.164** | **0.165** | **0.203** | **0.206** | **0.208** | **0.222** |  | **0.213** | **0.386** | **-0.124** | **-0.030** | **0.022** | **-0.090** | **-0.004** |
| *Habrotrocha pusilla nuda* | -0.005 | 0.93 | 0.486 |  | 0.005 | 0.006 | 0.022 | 0.044 | 0.063 | 0.065 | 0.069 |  | 0.056 | -0.145 | 0.018 | -0.091 | -0.146 | 0.062 | 0.216 |
| ***Habrotrocha spicula*** | **0.242** | **5.34** | **<0.001** |  | **0.097** | **0.156** | **0.172** | **0.189** | **0.265** | **0.298** | **0.298** |  | **-0.185** | **0.344** | **0.181** | **-0.214** | **-0.049** | **0.300** | **-0.387** |
| *Habrotrocha sylvestris* | -0.001 | 0.98 | 0.451 |  | 0.004 | 0.005 | 0.005 | 0.038 | 0.059 | 0.066 | 0.072 |  | 0.141 | -0.110 | 0.086 | -0.014 | -0.250 | 0.010 | 0.200 |
| *Habrotrocha tridens* | 0.033 | 1.46 | 0.191 |  | 0.000 | 0.020 | 0.072 | 0.096 | 0.098 | 0.101 | 0.104 |  | -0.136 | 0.156 | -0.119 | -0.180 | 0.136 | -0.155 | 0.119 |
| *Habrotrocha* sp. 1 | -0.026 | 0.66 | 0.707 |  | 0.018 | 0.018 | 0.018 | 0.021 | 0.025 | 0.049 | 0.050 |  | 0.150 | -0.046 | 0.061 | -0.124 | 0.078 | -0.024 | -0.128 |
| *Habrotrocha* sp. 2 | 0.059 | 1.85 | 0.087 |  | 0.021 | 0.027 | 0.033 | 0.037 | 0.126 | 0.128 | 0.128 |  | -0.203 | 0.316 | 0.055 | 0.005 | -0.004 | 0.253 | -0.237 |
| ***Habrotrocha* sp. 3** | **0.396** | **9.91** | **<0.001** |  | **0.259** | **0.259** | **0.431** | **0.431** | **0.434** | **0.441** | **0.441** |  | **-0.243** | **-0.224** | **-0.213** | **-0.244** | **0.184** | **0.168** | **0.162** |
| ***Macrotrachela ehrenbergii*** | **0.114** | **2.74** | **0.013** |  | **0.027** | **0.106** | **0.121** | **0.125** | **0.148** | **0.155** | **0.179** |  | **0.435** | **-0.185** | **0.065** | **0.059** | **-0.080** | **0.058** | **0.053** |
| *Macrotrachela habita* | -0.004 | 0.95 | 0.476 |  | 0.009 | 0.015 | 0.028 | 0.029 | 0.060 | 0.070 | 0.070 |  | -0.037 | 0.203 | -0.017 | 0.221 | -0.011 | 0.037 | -0.062 |
| *Macrotrachela insolita* | -0.018 | 0.76 | 0.619 |  | 0.001 | 0.004 | 0.015 | 0.035 | 0.046 | 0.054 | 0.057 |  | 0.075 | -0.128 | 0.034 | 0.043 | -0.246 | -0.029 | 0.128 |
| ***Macrotrachela musculosa*** | **0.103** | **2.56** | **0.019** |  | **0.044** | **0.099** | **0.118** | **0.118** | **0.120** | **0.121** | **0.169** |  | **0.025** | **-0.045** | **0.312** | **0.172** | **0.192** | **-0.164** | **-0.005** |

(cont.)

|  | ANOVA | | | | | | | |  | | | PLS R^2^ per latent factor | | | | | | | | | | | | | | | | | | | | |  | | | PLS scaled regression coefficients | | | | | | | | | | | | | | | | | | | | |
| --- | --- | --- | --- | --- | --- | --- | --- | --- | --- | --- | --- | --- | --- | --- | --- | --- | --- | --- | --- | --- | --- | --- | --- | --- | --- | --- | --- | --- | --- | --- | --- | --- | --- | --- | --- | --- | --- | --- | --- | --- | --- | --- | --- | --- | --- | --- | --- | --- | --- | --- | --- | --- | --- | --- | --- | --- |
| Species | Adj.R^2^ | | F | | | p | | |  | | | LF1 | | | LF2 | | | LF3 | | | LF4 | | | LF5 | | | LF6 | | | LF7 | | |  | | | Hphy | | | Psax | | | Psul | | | bark | | | Hphy:bark | | | Psax:bark | | | Psul:bark | | |
| ***Macrotrachela papillosa*** | | **0.108** | | | **2.64** | | | **0.016** | | |  | | | **0.052** | | | **0.065** | | | **0.076** | | | **0.076** | | | **0.165** | | | **0.170** | | | **0.174** | | |  | | | **-0.222** | | | **0.385** | | | **0.074** | | | **-0.051** | | | **0.097** | | | **0.174** | | **-0.286** |  |
| ***Macrotrachela plicata plicata*** | **0.079** | | **2.16** | | | **0.045** | | |  | | | **0.046** | | | **0.068** | | | **0.073** | | | **0.074** | | | **0.146** | | | **0.147** | | | **0.147** | | |  | | | **-0.195** | | | **0.388** | | | **0.036** | | | **-0.007** | | | **-0.052** | | | **0.158** | | | **-0.182** | | |
| *Macrotrachela plicata hirundinella* | 0.037 | | 1.52 | | | 0.171 | | |  | | | 0.029 | | | 0.036 | | | 0.043 | | | 0.069 | | | 0.075 | | | 0.095 | | | 0.108 | | |  | | | -0.067 | | | 0.198 | | | -0.004 | | | -0.047 | | | 0.112 | | | -0.294 | | | 0.154 | | |
| *Macrotrachela punctata* | -0.024 | | 0.69 | | | 0.683 | | |  | | | 0.012 | | | 0.034 | | | 0.038 | | | 0.040 | | | 0.041 | | | 0.051 | | | 0.052 | | |  | | | 0.133 | | | -0.043 | | | 0.104 | | | 0.042 | | | 0.021 | | | 0.116 | | | -0.102 | | |
| *Macrotrachela quadricornifera* | -0.030 | | 0.61 | | | 0.750 | | |  | | | 0.013 | | | 0.018 | | | 0.023 | | | 0.023 | | | 0.026 | | | 0.038 | | | 0.046 | | |  | | | -0.159 | | | -0.024 | | | -0.004 | | | -0.014 | | | 0.010 | | | -0.112 | | | 0.145 | | |
| *Mniobia bredensis* | 0.064 | | 1.92 | | | 0.076 | | |  | | | 0.014 | | | 0.055 | | | 0.055 | | | 0.119 | | | 0.122 | | | 0.133 | | | 0.133 | | |  | | | 0.136 | | | -0.100 | | | -0.172 | | | 0.023 | | | 0.316 | | | -0.095 | | | -0.015 | | |
| ***Mniobia incrassata*** | **0.461** | | **12.61** | | | **<0.001** | | |  | | | **0.165** | | | **0.257** | | | **0.310** | | | **0.452** | | | **0.457** | | | **0.498** | | | **0.501** | | |  | | | **-0.041** | | | **0.682** | | | **-0.307** | | | **-0.178** | | | **0.076** | | | **-0.315** | | | **0.118** | | |
| ***Mniobia magna*** | **0.107** | | **2.63** | | | **0.016** | | |  | | | **0.009** | | | **0.048** | | | **0.080** | | | **0.107** | | | **0.142** | | | **0.172** | | | **0.173** | | |  | | | **-0.174** | | | **-0.064** | | | **0.140** | | | **0.005** | | | **-0.104** | | | **0.216** | | | **-0.396** | | |
| *Mniobia montium* | 0.049 | | 1.70 | | | 0.119 | | |  | | | 0.000 | | | 0.027 | | | 0.048 | | | 0.115 | | | 0.116 | | | 0.119 | | | 0.119 | | |  | | | 0.190 | | | -0.030 | | | -0.118 | | | 0.110 | | | 0.242 | | | -0.169 | | | -0.073 | | |
| *Mniobia obtusicalcar* | -0.006 | | 0.92 | | | 0.491 | | |  | | | 0.010 | | | 0.040 | | | 0.044 | | | 0.059 | | | 0.060 | | | 0.068 | | | 0.069 | | |  | | | 0.086 | | | -0.099 | | | -0.095 | | | -0.014 | | | 0.206 | | | 0.010 | | | 0.009 | | |
| *Mniobia obtusicornis* | -0.016 | | 0.79 | | | 0.599 | | |  | | | 0.002 | | | 0.045 | | | 0.050 | | | 0.051 | | | 0.055 | | | 0.056 | | | 0.059 | | |  | | | 0.101 | | | -0.086 | | | 0.075 | | | 0.123 | | | 0.101 | | | -0.085 | | | 0.075 | | |
| *Mniobia recurvicornis* | 0.063 | | 1.91 | | | 0.077 | | |  | | | 0.088 | | | 0.100 | | | 0.115 | | | 0.117 | | | 0.117 | | | 0.127 | | | 0.132 | | |  | | | -0.178 | | | -0.164 | | | -0.034 | | | 0.113 | | | -0.085 | | | -0.077 | | | 0.048 | | |
| *Mniobia russeola* | 0.011 | | 1.14 | | | 0.343 | | |  | | | 0.008 | | | 0.022 | | | 0.022 | | | 0.027 | | | 0.051 | | | 0.068 | | | 0.083 | | |  | | | -0.203 | | | 0.089 | | | 0.200 | | | -0.070 | | | 0.075 | | | 0.112 | | | -0.281 | | |
| *Mniobia scabrosa* | 0.004 | | 1.06 | | | 0.397 | | |  | | | 0.036 | | | 0.067 | | | 0.070 | | | 0.070 | | | 0.078 | | | 0.078 | | | 0.078 | | |  | | | 0.197 | | | -0.024 | | | 0.102 | | | 0.050 | | | 0.037 | | | -0.064 | | | 0.063 | | |
| ***Mniobia scarlatina*** | **0.213** | | **4.68** | | | **<0.001** | | |  | | | **0.185** | | | **0.225** | | | **0.235** | | | **0.247** | | | **0.271** | | | **0.271** | | | **0.271** | | |  | | | **-0.075** | | | **0.380** | | | **0.183** | | | **-0.154** | | | **-0.131** | | | **0.123** | | | **-0.158** | | |
| *Mniobia tentans* | 0.072 | | 2.05 | | | 0.057 | | |  | | | 0.004 | | | 0.006 | | | 0.024 | | | 0.049 | | | 0.109 | | | 0.127 | | | 0.140 | | |  | | | 0.276 | | | -0.196 | | | 0.055 | | | 0.053 | | | -0.319 | | | -0.105 | | | 0.273 | | |
| *Mniobia* sp. 1 | 0.014 | | 1.20 | | | 0.314 | | |  | | | 0.011 | | | 0.055 | | | 0.063 | | | 0.065 | | | 0.078 | | | 0.085 | | | 0.087 | | |  | | | 0.180 | | | -0.183 | | | 0.191 | | | 0.047 | | | 0.075 | | | -0.032 | | | -0.003 | | |
| *Mniobia* sp. 2 | 0.007 | | 1.10 | | | 0.370 | | |  | | | 0.038 | | | 0.045 | | | 0.058 | | | 0.077 | | | 0.077 | | | 0.080 | | | 0.081 | | |  | | | 0.055 | | | -0.142 | | | -0.135 | | | 0.091 | | | 0.141 | | | -0.063 | | | -0.060 | | |
| ***Otostephanos torquatus*** | **0.087** | | **2.30** | | | **0.034** | | |  | | | **0.012** | | | **0.015** | | | **0.015** | | | **0.110** | | | **0.131** | | | **0.136** | | | **0.155** | | |  | | | **-0.039** | | | **-0.190** | | | **0.396** | | | **0.004** | | | **-0.171** | | | **-0.003** | | | **0.155** | | |
| ***Pleuretra lineata*** | | **0.079** | | **2.17** | | | **0.045** | | |  | | | **0.025** | | | **0.035** | | | **0.051** | | | **0.056** | | | **0.127** | | | **0.145** | | | **0.147** | | |  | | | **-0.170** | | | **0.469** | | | **-0.149** | | | **0.049** | | | **-0.037** | | | **0.101** | | **-0.032** | | |
| ***Pleuretra* sp. 1** | | **0.103** | | **2.56** | | | **0.019** | | |  | | | **0.018** | | | **0.018** | | | **0.020** | | | **0.077** | | | **0.160** | | | **0.161** | | | **0.169** | | |  | | | **-0.100** | | | **0.238** | | | **0.083** | | | **0.088** | | | **-0.221** | | | **0.406** | | **-0.139** | | |
| *Philodina plena* | 0.004 | | 1.06 | | | 0.397 | | |  | | | 0.066 | | | 0.067 | | | 0.073 | | | 0.077 | | | 0.077 | | | 0.078 | | | 0.078 | | |  | | | -0.060 | | | -0.133 | | | -0.127 | | | 0.054 | | | 0.044 | | | -0.037 | | | -0.036 | | |
| *Philodina proterva* | 0.068 | | 1.99 | | | 0.066 | | |  | | | 0.130 | | | 0.131 | | | 0.131 | | | 0.132 | | | 0.134 | | | 0.134 | | | 0.137 | | |  | | | -0.119 | | | -0.217 | | | -0.113 | | | 0.030 | | | 0.093 | | | -0.021 | | | 0.073 | | |
| *Philodina rugosa* | *0.030* | | *1.43* | | | *0.205* | | |  | | | *0.038* | | | *0.051* | | | *0.056* | | | *0.059* | | | *0.086* | | | *0.095* | | | *0.102* | | |  | | | *0.157* | | | *0.103* | | | *-0.066* | | | *-0.135* | | | *-0.119* | | | *-0.186* | | | *0.194* | | |
| *Philodina vorax* | 0.062 | | 1.90 | | | 0.079 | | |  | | | 0.092 | | | 0.105 | | | 0.105 | | | 0.114 | | | 0.114 | | | 0.116 | | | 0.131 | | |  | | | -0.243 | | | -0.224 | | | 0.071 | | | -0.024 | | | 0.018 | | | 0.017 | | | -0.059 | | |
| *Scepanotrocha* sp. 1 | 0.008 | | 1.11 | | | 0.361 | | |  | | | 0.005 | | | 0.021 | | | 0.023 | | | 0.046 | | | 0.069 | | | 0.081 | | | 0.081 | | |  | | | 0.125 | | | -0.134 | | | 0.145 | | | 0.069 | | | -0.154 | | | -0.048 | | | 0.227 | | |
